# Barriers and facilitators to attending postpartum diabetes screening among women with previous gestational diabetes in China: A qualitative analysis

**DOI:** 10.1111/dme.70043

**Published:** 2025-04-11

**Authors:** Jing Huang, Rita Forde, Angus Forbes, Judith Parsons

**Affiliations:** ^1^ Division of Care in Long‐Term Condition King's College London London UK; ^2^ School of Nursing and Midwifery University College of Cork Cork Ireland

**Keywords:** gestational diabetes, qualitative methods, screening, type 2 diabetes

## Abstract

**Introduction:**

Gestational diabetes mellitus (GDM) is a common complication of pregnancy associated with a higher risk of developing type 2 diabetes (T2DM) in the future. Postpartum diabetes screening is important to identify glucose intolerance and introduce diabetes prevention support. However, screening uptake is suboptimal, including in China where the prevalence of GDM is high. There is limited evidence on the barriers and facilitators to screening uptake among Chinese women.

**Aims:**

To explore the barriers and facilitators of postpartum diabetes screening uptake among Chinese women with GDM to inform the development of an intervention to increase screening attendance.

**Methods:**

Women with current or previous GDM were recruited from social network platforms and pregnancy groups in China. Data were collected using semi‐structured interviews and analysed using Framework Analysis to identify themes related to the barriers and facilitators for screening uptake.

**Results:**

Twenty‐four women with current (*n* = 4) or previous (*n* = 20) GDM participated. The postpartum glucose screening attendance rate was 35% among those with previous GDM. Screening uptake was influenced by: risk awareness of T2DM and its complications, interactions with healthcare providers (HCPs), screening‐related factors (understanding and beliefs, accessibility and acceptability of the test) and motivation to maintain personal health, which was influenced by fear of T2DM, lack of symptoms, confidence in self management without support, and prioritisation of the child's needs.

**Conclusion:**

Postpartum screening uptake after GDM could be boosted through raising risk awareness, more constructive communication from HCPs, increasing the acceptability and accessibility of screening procedures, and addressing psychological factors related to attendance.

**What‘s new?**

**What is already known?**

- Postpartum diabetes screening is usually recommended to identify early glucose intolerance and introduce timely diabetes prevention strategies.
- However, uptake is suboptimal, especially in China where gestational diabetes (GDM) is prevalent.

**What this study has found?**

- Risk awareness, screening‐related factors, interactions with healthcare providers and motivation to maintain personal health were closely related to the uptake of postpartum diabetes screening among Chinese women with GDM.

**What are the implications of this study?**

- Postpartum screening uptake among women with previous GDM could be boosted through: raising risk awareness, effective communication with HCPs, increasing the acceptability and accessibility of the screening procedure, as well as addressing psychological factors related to attendance.

## 1 INTRODUCTION

Gestational diabetes mellitus (GDM) is a common pregnancy complication. China is one of the countries with a high prevalence of GDM. Estimations of current prevalence rates in China range from 14.8% to 24.7% of pregnancies.^1^, ^2^, ^3^, ^4^, ^5^ This prevalence is rising, following the increasing proportion of pregnant women with obesity and advanced maternal age.^1^, ^6^, ^7^ GDM is associated with multiple negative impacts on both the mother and the new‐born, including an increased risk of perineal trauma, shoulder dystocia, cesarean section, neonatal hypoglycaemia, respiratory distress syndrome and admissions to neonatal intensive care.^8^, ^9^, ^10^, ^11^, ^12^, ^13^ While most women return to normoglycaemia after childbirth, a high proportion progress to Type 2 Diabetes Mellitus (T2DM).^14^ Women with GDM have a 10‐fold higher risk of developing T2DM, with the incidence peaking within 5 years of childbirth.^14^ The recent findings from the hyperglycaemia and adverse pregnancy outcome (HAPO) follow‐up study reported that more than half of women with previous GDM developed impaired glucose regulation.^15^ GDM is also a risk factor for GDM in future pregnancies.^16^

Given the long‐term impacts of GDM, postpartum care for women with previous GDM is important, and postpartum diabetes screening is recommended in most guidelines.^17^, ^18^, ^19^, ^20^ A 2‐h 75 g oral glucose tolerance test (OGTT), which involves the consumption of a 75 g glucose solution and blood samples at one‐ and two‐hour intervals, is recommended as the standard screening method for postpartum diabetes screening in most current guidelines.^17^, ^21^, ^22^, ^23^ Guidelines in China, developed by the Chinese Diabetes Society and the Diabetology Committee of the Chinese hospital Association, also recommend a 2‐h 75 g OGTT for women with GDM 4 to 12 weeks postpartum.^24^, ^25^ In some countries, other methods for screening are utilised. The National Institute for Health and Care Excellence (NICE) guidelines in the UK do not recommend routine OGTT for postpartum screening in women with GDM. They recommend a fasting plasma glucose test (FPG) or a glycated haemoglobin test (HbA1c).^19^

Screening is important in identifying either: a return to normoglycaemia, impaired glucose tolerance or diabetes. Screening also provides an opportunity to help women better understand their diabetes risks and introduce preventative lifestyle interventions to reduce the risks of recurrent GDM and T2DM. However, despite these guidelines and the evidence supporting the importance of screening, a significant proportion of women do not access or attend screening in many parts of the world. Previous studies indicated that less than half of women attend postpartum screening.^26^, ^27^, ^28^ Furthermore, nonattendance at screening has been associated with a higher conversion rate to T2DM.^29^

Therefore, recognising the possible barriers and facilitators of women's participation in postpartum diabetes screening is important to inform the development of behavioural interventions to enhance screening uptake in this population. Previous studies^30^, ^31^, ^32^, ^33^ have reported a range of reasons for suboptimal screening uptake. These include individual barriers, such as women's risk perception of GDM and competing priorities in the postpartum period; practice‐level barriers, such as inadequate information and education from healthcare professionals (HCPs); and policy‐level factors, such as the type of screening methods recommended and the introduction of GDM registers. However, the majority of these studies were conducted in Western countries where the healthcare systems and sociocultural contexts are very different from China. Hence, it is important to explore current barriers to GDM screening uptake in the Chinese context. There is a paucity of data on the factors that may influence women's attendance at postpartum diabetes screening. Available evidence indicates a low screening uptake among Chinese women with GDM, with an attendance rate ranging from 13.1% to 38.4%,^34^, ^35^, ^36^ suggesting the need to develop an effective and culturally tailored intervention.

To address the current deficit in understanding the barriers to screening uptake in China, we undertook a qualitative study to explore women's experiences and views on postpartum diabetes screening in depth. This approach allowed us to elicit information on the barriers and facilitators that women experience, thereby enabling us to explicate potential mechanisms to be incorporated into interventions to improve screening uptake.

Taken together, the aim of this qualitative study was to explore the barriers and facilitators of attending postpartum diabetes screening among women with previous GDM in the Chinese context, to inform a theoretical framework for the development of interventions to improve screening uptake.

## 2 METHODS

### 2.1 Study design

The qualitative study used online semi‐structured interviews with women with current or previous GDM to explicate the barriers and facilitators to the uptake of postpartum diabetes screening. The barriers and facilitators identified were then mapped onto the Theoretical Domains Framework (TDF) and Capability, Opportunity, Motivation‐Behaviour (COM‐B) model to provide a theoretical structure of the modifiable factors influencing screening uptake, against which intervention strategies targeting those factors could be identified. The TDF incorporates 14 domains from 33 behaviour change theories.^37^, ^38^ The TDF domains can be mapped onto the Behaviour Change Wheel and COM‐B model to provide a comprehensive model of targetable behavioural mechanisms to inform intervention design.^39^

The reporting of this study followed the Consolidated criteria for Reporting Qualitative research (COREQ).^40^

### 2.2 Participants

Women with previous gestational diabetes were purposively recruited based on the following eligibility criteria: (1) a self‐reported hospital‐confirmed diagnosis of GDM; (2) currently experiencing a GDM pregnancy or previous GDM within the last 5 years; (3) aged >18 years; (4) able to speak and understand Chinese; and (5) have access to internet‐connected devices (phone, laptop, or computer). Women were excluded if they had pre‐existing Type 1 or Type 2 Diabetes Mellitus or severe mental illness, or reduced capacity to provide informed consent. Eligible women were recruited through local pregnancy groups, which are typically organized by women themselves for the purpose of sharing relevant pregnancy‐related information, as well as via Chinese social media platforms (e.g. WeChat). An electronic form of written consent was obtained, and interviews were then scheduled for the eligible participants. The sample size of this study was determined by information power.^41^ Five key aspects were considered to evaluate the sufficiency of information power based on: the aims of the study, the sample specificity, theoretical background, quality of dialogue, and the model for the analysis. According to the principles of information power, studies with narrow study aims, participants highly aligned to the study aims, strong theoretical support, focused interview dialogue, and in‐depth narratives or discourse analyses typically require fewer participants. Information power was continuously assessed throughout the data collection process and deemed sufficient after conducting 24 semi‐structured interviews.

### 2.3 Data collection

An interview guide (see File S1) was developed by the research team based on previous literature related to the research topic. This guide was modified by two senior researchers and pilot tested by two participants who had prior GDM, with no subsequent modifications. The semi‐structured interviews were conducted online to provide a flexible and convenient interview context for the participants who were either pregnant or mothers busy with childcare.

Before the interviews, a short electronic form was sent to the participants to collect socio‐demographic information, including postpartum stage, age, educational background, marital status, profession, number of pregnancies and childbirths, insulin injections during pregnancy, attendance at postpartum diabetes screening, and the screening outcomes (for postpartum women). The attendance at postpartum diabetes screening in this study referred to taking an oral glucose tolerance test (OGTT) within 4–12 weeks postpartum, in accordance with current screening guidelines in China.^24^ Participants who undertook other types of glucose tests (e.g. fasting glucose test) or did an OGTT >12 weeks postpartum were categorised as not attending screening. Once the interview was completed, participants received an e‐voucher (50RMB) for their contribution. Each interview was conducted by the same researcher (JH) to ensure consistency.^42^ All interviews were audio‐recorded, transcribed verbatim and anonymised.

### 2.4 Data analysis

Transcribed interviews were analysed using thematic Framework Analysis.^43^ Following this approach, the analysis was conducted in five integrated phases: (1) Familiarisation: Following transcription, the interviewer repeatedly listened to the audio recordings and reviewed the transcripts. (2) Identifying a thematic framework: Firstly, a sample of transcripts was inductively coded to generate an initial framework for organising the data. During this phase, key concepts and categories were identified, and the framework was organised into a hierarchical structure, including the main themes and sub‐themes. (3) Sorting/indexing: All transcripts were coded to the framework, which was expanded and refined iteratively through discussions with the research team. (4) Charting: Data linked to the original transcript for context were summarised and tabulated within the analytic framework. (5) Mapping and interpretation: The framework was reviewed by the research team, data were grouped, and key dimensions were identified, resulting in the themes and subthemes as presented in the findings section.

The interviews were conducted in Chinese by JH. The primary data analysis was performed, and illustrative quotes and themes were translated by the interviewer (JH, who is a native Chinese speaker). These analysis results were then cross‐checked, reviewed, and discussed collectively by multiple researchers (*n* = 3) in the research team to ensure consistent coding. An audit trail of coding decisions and framework revisions was kept to enhancing reliability and transparency.

### 2.5 Reflexivity

The interviewer JH (MSc, female) has prior experience as an intern midwife and has provided care for women with GDM. Other members of the research team have clinical and academic backgrounds in diabetes and pregnancy and extensive experience in conducting research related to GDM and qualitative studies.

### 2.6 Ethics

Ethical approval for this study was granted by the Research Ethics Office of King's College London (Approval number: LRS/DP‐22/23‐35852, date: 10‐07‐2023). This study followed the guidelines of the Declaration of Helsinki. Informed consent was obtained from each participant before the interview.

## 3 FINDINGS

Twenty‐four women with a mean age of 33 years (range 27–43 years) participated. Four women were pregnant at the time of the interview, and the remainder ranged from 2 months to 5 years postpartum. About 83.3% (*n* = 20) of the participants were postpartum women. Among the postpartum women, 65.0% (*n* = 13) did not attend postpartum diabetes screening, and 30.0% (*n* = 6) reported abnormal glucose levels measured by various tests (including self monitoring capillary glucose test results at home and a fasting glucose/OGTT test in the hospital). About 66.7% (*n* = 16) of the participants were primiparous women. Additionally, 16.7% (*n* = 4) of participants used insulin during pregnancy. The detailed characteristics of participants are presented in Table 1.

**TABLE 1:** Socio‐demographic characteristics of participants (*n* = 24).

Socio‐demographic variables	*n* (%)
Age	
25–29	4 (16.7)
30–34	12 (50.0)
35–39	5 (20.8)
≥40	3 (12.5)
Educational level	
High school	3 (12.5)
Vocational college degree	7 (29.2)
Bachelor's	10 (41.7)
Master's or above	4 (16.7)
Marital status	
Married	24 (100.0)
Employment status	
Full‐time job	19 (79.1)
Housewife	5 (20.8)
Number of childbirths	
0	4 (16.7)
1	12 (50.0)
2	8 (33.3)
Number of pregnancies	
1	16 (66.7)
2	7 (29.2)
3	1 (4.1)
Number of GDM pregnancies	
1	23 (95.9)
2	1 (4.1)
Medication use during pregnancy	
Insulin	4 (16.7)
None	20 (83.3)
Attendance at postpartum diabetes screening among postpartum women	
Yes	7 (35.0)
No	13 (65.0)
Postpartum glucose levels measured by any glucose tests	
Normal	8 (40.0)
Abnormal	6 (30.0)
Not known	6 (30.0)
Stage	
Antenatal period	4 (16.7)
6 weeks to 6 months postpartum	5 (20.8)
7–12 months postpartum	8 (33.3)
More than 1 years to 2 years postpartum	4 (16.6)
More than 2 years to 5 years postpartum	3 (12.5)

### 3.1 Themes and sub‐themes

**FIGURE 1:**
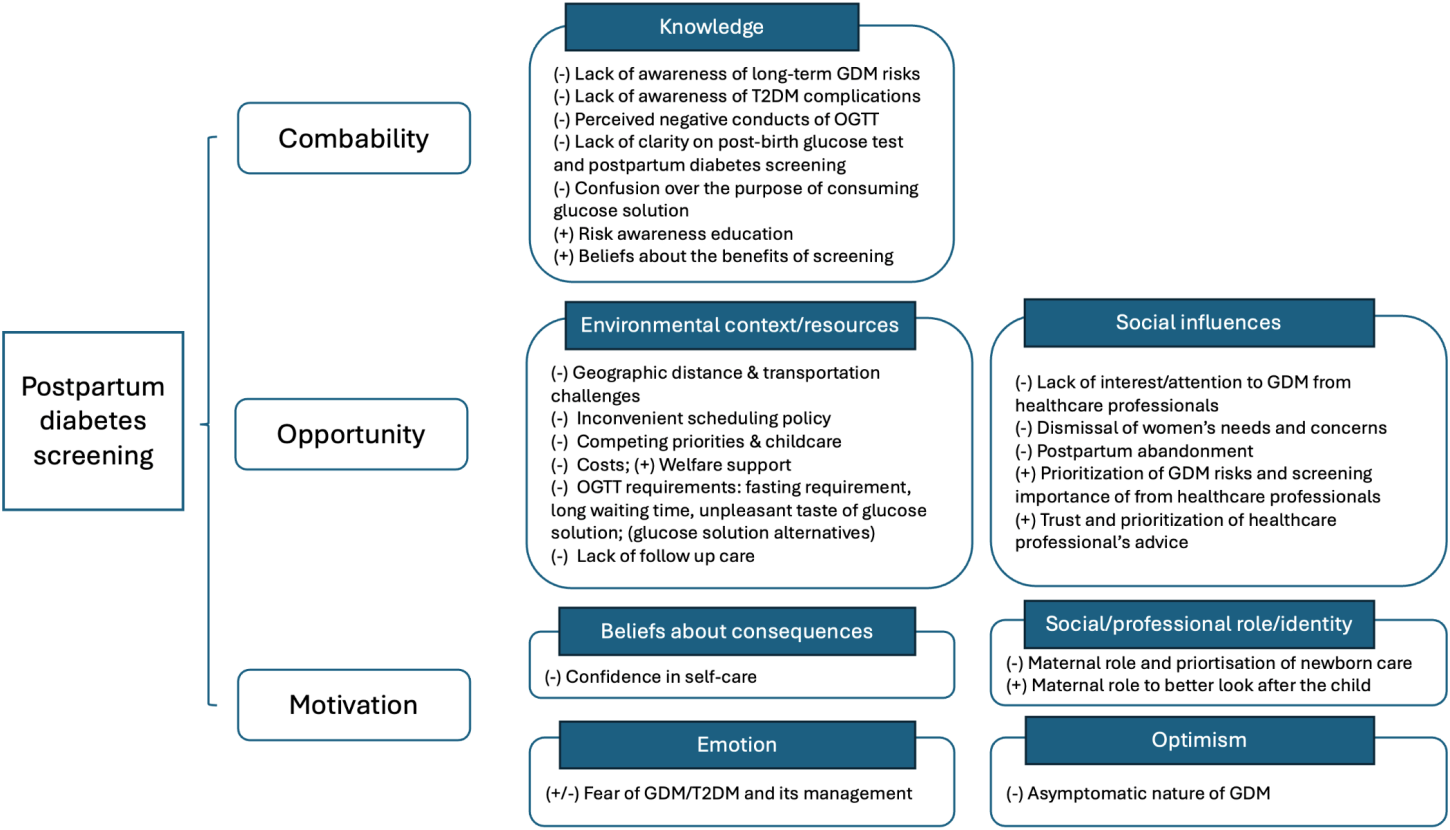
Barriers and facilitators to postpartum diabetes screening and the theoretical domain (TDF) framework and the COM‐B model. (−) barrier; (+) facilitators.

Four themes and seven subthemes were generated from the analysis. These are described below and summarised with relevant quotes from the data in Table 2. The barriers and facilitators were then mapped into the TDF framework and COM‐B model (see Figure 1).

**TABLE 2:** Themes, sub‐themes and illustrative quotes.

Themes, sub‐themes and quotes
Theme: Risk awareness
*Awareness of long‐term GDM risks* “After giving birth, it (glucose level) will also come down, so there won't be much anxiety about the possibility of developing diabetes. It won't cause too much anxiety for myself, right after giving birth, the blood glucose would return to a normal state”. P22‐postpartum (2 m)—non‐attendee “… I find it strange because some people say that after giving birth, they have diabetes”. P16‐postpartum (2 yrs)—non‐attendee “For me, it's definitely not necessary. I'm not giving birth anymore, so it doesn't matter to me. That's how I see it”. P16‐Postpartum (2 yrs)—non‐attendee *Awareness of T2DM complications* “Since I was little, I have known that my grandaunt has likely had diabetes for many years. It progressed to the point where she (the participant's grandaunt) eventually lost her eyesight, and ultimately, complications from diabetes led to her passing away. So, I am aware that diabetes is a very serious matter. However, many people may not have someone with related health issues around them, or they may not pay attention, and thus, they are unaware of how truly frightening it can be. As a result, they may not be concerned about it.” P18‐postpartum (2 m)‐attendee *Risk awareness education* “Another aspect is that the knowledge promotion should be comprehensive, covering why it's essential to undergo such screenings and understanding the potential risks. In modern times, people are generally concerned about their health, especially after childbirth when the body undergoes significant changes. So, if there is effective and informative promotion, I believe many would be willing to participate. At least, I would be willing to do so”. P10‐Pospartum (9 m)—attendee

Theme: Screening‐related factors
*Sub‐theme: Understanding and beliefs on screening methods, procedures and timing* *Perceived negative conducts of screening* “If the glucose solution is too sweet, could it be particularly harmful to my baby? I'm worried about this… After all, having had diabetes before, if I drink a bowl of sweetened water, I fear it might not be good for the baby, as I've heard that it could be harmful to the baby… If it's delivered through breast milk, it might not be good for the baby”. P2‐Postpartum (8 m)—non attendee “So, because drinking the glucose solution could be quite damaging to the pancreas, I decided not to drink it when I reached the 42nd day”. P8‐Postpartum(1yr5m)‐non‐attendee “They (HCPs) suggested that it's better to do it after giving birth, just to be cautious. But I think the method of using an oral glucose tolerance test, which involves consuming sugary drinks, may not be very good for my pancreas, not very friendly. Therefore, I haven't done it”. P13‐Postpartum (1yr5m)‐non‐attendee. “Think about it, they draw several tubes of blood from me. How much do I have to eat to replenish that?” P16‐Postpartum (2 yr)‐non‐attendee *Lack of clarity between post‐birth glucose test and postpartum screening* “After I was discharged, at that time, following the caesarean section, there should be a period of about 2 to 3 days where the diet is not normal. After testing the blood glucose, it was found to be normal, and the doctor confirmed that the blood glucose levels were normal…At that time, I naturally ate less, and my blood glucose levels were within the normal range when tested. Therefore, we overlooked this issue…”. P4‐Postpartum(4yr8m)‐non‐attendee “After giving birth to my daughter, on the third day, the doctor tested and found that my blood glucose levels had returned to normal. After that, I didn't pay much attention to this aspect”. P5‐Postpartum (10 m)‐non‐attendee “However, the doctor said to come back for a blood glucose check one month after discharge, but I tested it at home and it was normal, so I didn't go to the hospital…If, like in my case, the result was only slightly above normal, and it returned to normal after giving birth, I feel that the necessity of participating in screenings is not very high”. P20‐Postpartum (4 yrs)‐not‐attendee *Confusion over the purpose of consuming glucose solution* “I feel that drinking such sweet glucose solution, can the blood glucose results obtained from it be accurate?”. P11‐Postpartum (1 yr)‐non‐attendee “If I eat something sweet, you know it will definitely be high after an hour”. P16‐Postpartum (2 yrs)‐non attendee “When I had gestational diabetes, I noticed that many people had similar questions when searching for information online…Glucose tolerance, what is it exactly? Many people are unclear about this. You'll find that some individuals question why they are asked to drink a sugar solution and have their blood glucose tested, especially if they don't typically consume very sweet items. Their point is that they don't usually eat such sweet things, so their blood glucose shouldn't be that high… I've also been confused, wondering if drinking so much sugar would make my blood glucose high. I thought that by not consuming as much sugar, my levels might not be high”. P18‐Postpartum (3 m)‐attendee *Beliefs on the benefits of screening* “I don't like drinking glucose solution either…I have no choice but to attend it, it seems like this test is more accurate, right?”. P12‐Postpartum (2 yr)‐attendee. “It is essential (to take the test). For example, if any of your indicators exceed the normal range during pregnancy, it's necessary to schedule a follow‐up appointment postpartum to check whether you have recovered”. P1‐Antepartum (37w)‐NA. “I would rather go and drink the glucose solution, let the doctor see how to control and if medication is necessary. Because the medication is also based on your blood glucose situation, the doctor cannot just prescribe medication without considering your specific blood glucose levels”. P21‐Postpartum (3 m)‐attendee
*Sub‐theme: Accessibility of the test* *Geographic distance & transportation challenges* “From our village to the city, if you have a car and can drive, you might drive there yourself. However, if you have to take a bus while carrying a child, it will take more than an hour, and there was no time left for the test”. P5‐Postpartum (10 m)‐non‐attendee “However, it was inconvenient as the place was too far away, and I couldn't go far while taking care of the child on my own…For me, taking a day off, traveling by bus for the check‐up, and then spending money on it. I don't think it's necessary.” P11‐Postpartum (1 yr)‐non‐attendee *Scheduling policy* “He/she (the obstetrician) didn't prescribe it for me; instead, he/she (the obstetrician) suggested that I see an endocrinologist (to get the test). I found it troublesome, so I didn't go…”. P13‐Postpartum (1yr5m)‐non‐attendee *Competing priorities & childcare* “I have to carry my child up and down the stairs, and then go to each department. It takes several hours to finish, and then I really don't have time, and I still need to fast for the test.” P11‐Postpartum (1 yr)‐non‐attendee. “Because there was also a 42‐day check‐up for the baby, and I usually take care of him myself, so I felt that I didn't have time, and I didn't go”. P13‐Postpartum (1yr5m)‐non‐attendee. *Costs* “Some people don't have medical insurance, and a casual visit to the hospital can cost a few hundred yuan. If all the test results are normal during the time of childbirth, why waste this money? You have to consider the practical aspects like money, time, and willingness. If it's free, maybe around 60% of people would be willing. However, if they have to pay, some people, after giving birth, may not even go for the postpartum check‐up after 42 days”. P16‐Postpartum (2 yrs)‐non‐attendee “I feel that financial factors are undoubtedly a reality. If, for example, there could be a way to make it more accessible, like offering free glucose tolerance screenings for mothers within the first year postpartum, it could help encourage more participation”. P10‐postpartum (9 m)‐attendee 3. “It still depends on whether the policies and welfare support are in place because many factors, such as family situations, make it difficult for many mothers. To be honest, the pressure on mothers, especially the younger generation, is significant. There is a lot of stress in their lives, and many people face challenging living conditions. It's not as simple as just taking the initiative. Definitely, if national policies or welfare measures are more comprehensive, it might be easier for mothers. If they have to spend time and money on these tests, I believe many mothers wouldn't be able to manage it”. P9‐Postpartum (5 yr)‐attendee
*Sub‐theme: Acceptability of the test* *Fasting requirement, waiting time and taste of glucose solution* “And really don't want to come for the second time, it's really uncomfortable… Drinking sugar makes me want to vomit…Drinking the glucose solution was difficult for me. Also, because my stomach wasn't well, I had a few hard times when I fainted or felt like I was in shock due to discomfort. It was very unpleasant, and I'm afraid of the possibility of low blood glucose on an empty stomach…”. P13‐Postpartum (1yr5m)‐not‐attendee “Yes, after all, to test for postprandial glucose level, you have to wait for quite a while. It takes a whole morning, right? Because you have to add the time for registration and all … It feels like going early in the morning on an empty stomach, and it goes on until around 10:00 or 11:00. So, I didn't participate”. P15‐Postpartum (11 m)‐non‐attendee “You have to wait for a long time, after drinking glucose solution, you have to go for a test again after an hour. Then, if you go in the morning at 8:00, you can only wait until 11:30…. I haven't taken a postpartum OGTT test, I don't want to drink glucose solution, I measured fasting blood glucose… It's too sweet, the glucose solution I had before was too sweet…”. P19‐Postpartum (8 m)‐non‐attendee “Because I feel that drinking glucose solution, well, from my own experience during pregnancy, it was quite painful”. P24‐Postpartum (9 m)‐not‐attendee *Glucose solution alternatives* “Another option is to eat white steamed buns. If the correlation with white steamed buns and its effects on glucose levels is well researched, it could be a substitute. Compared to consuming a large amount of glucose solution, this option may be more acceptable”. P14‐Anterpartum(26w)‐NA “However, the process of drinking glucose solution is quite painful; it's really unpleasant because it's too sweet. I've seen in some places they introduced a glucose tolerance bun, so you eat a bun on an empty stomach for the glucose tolerance test. I might find that more acceptable with a lower resistance level”. P23‐postpartum (2 m)‐attendee “I think the method of eating steamed buns can be promoted to various obstetrics and gynecology hospitals. Because originally, on an empty stomach, you feel very hungry. Eating steamed buns gives you a feeling of fullness, so after eating, you won't feel as hungry and uncomfortable. In contrast, after drinking the sugar water, there is no such feeling, and I still feel very hungry…If they eat steamed buns, I think the level of suffering might be slightly lower than drinking sugar water”. P18‐postpartum (2 m)‐attendee

Theme: Interactions with HCPs
*Sub‐themes* *Lack of Interest and attention to GDM and screening from HCPs* “It seems that I had to proactively inquire about these matters (GDM management); the doctor would only discuss them when prompted. I don't know about other doctors, but I assume it might be a common approach because they deal with so many pregnant women every day”. P1‐Antepartum (37w)‐NA “Actually, the doctor just dismissed me with a few words, didn't ask me to check anything, and there was no further follow‐up. I even asked if I should have another re‐examination, but they said it wasn't necessary. So, I haven't paid much attention, but I am still conflicted. I fear that later on, as I read online, there might be a risk of developing type 2 diabetes in the future…They didn't tell me to do it, and they didn't explain how to monitor the condition later. It was only later when I read online about the potential progression to type 2 diabetes that I started paying attention. However, I feel like the doctors in the hospital don't seem particularly concerned… When I asked if there's a risk of developing type 2 diabetes as many online sources suggest, they didn't provide a clear response. In the end, they just said I didn't need to check, so I didn't”. P2‐Postpartum (8 m)‐non‐attendee “After giving birth, she (the obstetrician) said, “Your blood glucose is normal, so there's no problem,” and then she (the obstetrician) didn't provide any instructions or precautions for after going home…If the doctor had reminded me to undergo the glucose tolerance test at that time, I would have done it. I would definitely have gone for it because health comes first, right? Yes. So, since the obstetrician didn't mention it at all, I overlooked it”. P4‐Postpartum (4yr8m)‐non attendee “If the doctor had emphasized and educated me more about this matter at that time, there's a possibility that I would have promptly gone for the check‐up to confirm if it was type 2 diabetes”. P5‐Postpartum(10 m)‐not‐attendee *Dismissal of women's concerns and needs* “Later on, I took the initiative to ask, but was told that I don't need to worry about it after giving birth. I felt confused, so I resorted to searching online to figure out what to do. Feeling anxious, I discovered many people facing similar issues, which added to my panic. I also asked at the hospital, but the doctors said there's no need for further examination and didn't provide guidance on what steps to take…”. P2‐Postpartum (8 m)‐non‐attendee *Postpartum abandonment* “During my hospital stay, the doctors, families, and nurses all focused on the baby, and no one even informed me about the significant risks associated with gestational diabetes”. P5‐Postpartum (10 m)‐non‐attendee *Prioritization of GDM risks and screening importance* “My doctor is a very good one, but many others haven't been informed by their doctors. They just mention that your blood glucose is high, and that's it. Yes, and from my observations in the group, most people seem unaware of the risks”. P18‐Postpartum (2 m)‐attendee “Even though the risks are often relatively low, considering the impact on the fetus and one's own well‐being, most people would still choose to control it. Hospitals (HCPs) tend to present these issues in a rather alarming manner”. P7‐Postpartum (8 m)‐attendee “Because here, when we were discharged, they provided a list and informed us that on the day of discharge, during the doctor's rounds, they reminded us to come back for a follow‐up check after 42 days”. P21‐postpartum (3 m)‐attendee “After giving birth, the hospital actually scheduled a postpartum follow‐up for me, which included a glucose tolerance test as part of the examination… I actually don't want to drink (glucose solution), but the problem is the test was already scheduled for me” P15‐Postpartum (11 m)‐non‐attendee *Trust and prioritization of HCP's advice* “I don't really have any resistance. If the doctor advises me to undergo a check‐up, regardless of what it is, I believe that the doctor is always looking out for my well‐being and the well‐being of my child. Whatever the doctor recommends, I just follow their process. I wouldn't refuse what the doctor suggests”. P21‐Postpartum (3 m)‐attendee *Lack of follow up care* “I think what it means is that if I get my blood glucose tested and it's abnormal, right? However, at the hospital, they don't have any better advice for you. They just advise you to be careful about what you eat”. P6‐Postpartum (6 m)‐non‐attendee “Because many people, after discovering high blood glucose, don't take further action. Some doctors may inform you that your blood glucose is high, and women might not know what to do next, so it just ends there…”. P18‐Postpartum (2 m)‐attendee

Theme: Motivation to maintain personal health
*Sub‐themes* *Fear* “If everyone goes online, they may come across some news related to the glucose tolerance test. They might feel that if the test results are not good, it means controlling diet and even having to prick fingers. This is something that many people are afraid of”. P18‐Postpartum (2 m)‐attendee “I'm thinking that if I have diabetes, it would be troublesome. If I develop diabetes after childbirth, it will feel like someone who loves eating sweets but can't enjoy them anymore. Not being able to eat sugar or sweet things would make me feel uncomfortable and take away the joy”. P13‐Postpartum (1yr5m)‐non‐attendee “Another aspect is that she (women with GDM) is too scared and doesn't want to get tested because if the test result is abnormal, she (women with GDM) might have to control her diet”. P14‐Antepartum (27w)‐NA “Why do you think I was concerned about it for a while after giving birth? I was worried that if my blood glucose level didn't decrease, wouldn't I be at risk of getting diabetes?” P12‐Postpartum (2 yr)‐attendee *Looking after the child* “She (women with GDM) started paying attention, whether you consider it a psychological strategy or not, I don't know. Personally, I think, with a small baby, a mother's instinct is not to have any health issues. It's a natural reaction because you have to take care of the baby. When you're sick, you may not care if there's no one to look after you, but when your health is compromised and you need to take care of the baby, it becomes a concern. How do you take care of the baby in that situation”. P9‐Postpartum (5 yr)‐attendee “She (women with GDM) might simplify it as a pregnancy‐related complication, but if you monitor it for an extended period after childbirth, you can pinpoint the exact reasons for your diabetes—whether it's genetic, lifestyle habits, or other factors. From a genetic perspective, if the genetic connection is significant, she (women with GDM) would be more inclined to observe her child's risk of developing diabetes in the future… This motivation would drive her to thoroughly understand her condition, leading her to undergo screenings…” P14‐Antepartum(26w)‐NA “People around me, including those in our group, after giving birth with gestational diabetes, they do pay attention for a period of time. However, I think as time goes on, everyone may forget, and still focus more on the children”. P15‐Postpartum (11 m)‐non‐attendee “After giving birth to the child, for a while, I would also worry about my own health. However, being busy taking care of the child and cooking for myself, I eventually forgot about it”. P11‐Postpartum (1 yr)‐non‐attendee “To be honest, after giving birth to the baby, I felt relieved, but before having the baby, I was indeed worried”. P11‐Postpartum (1 yr)‐ non‐attendee *Confidence in self care* “The reason I haven't gone for testing is because I am very careful about what I eat now; I don't eat randomly … I have always been cautious about my diet. I avoid foods that can cause an increase in blood glucose levels, such as fried foods or other items that are generally not good for health”. P6‐Postpartum (6 m)‐non‐attendee “I wasn't worried because I had been consistently controlling my diet. I cooked my own meals, consuming less oil and salt…I wasn't particularly anxious; I simply controlled my diet on my own”. P8‐Postpartum (1yr5m)‐not‐attendee “Because I feel like I've been controlling it well. Throughout my pregnancy, I relied on diet, and I believe I've managed my blood glucose very well… so I didn't go for the OGTT again”. P15‐Postpartum (11 m)‐non‐attendee *Asymptomatic nature of hyperglycaemia* “Like the doctor told me, blood glucose does have a deceptive nature because high blood glucose doesn't seem to have any immediate reactions. There won't be any stomach aches or headaches; you won't feel anything. The potential risks might take a long time to surface, and you won't perceive anything in between. People tend to think, ‘I don't feel anything, so why bother?’ Many people have this reaction. My obstetrician told me that when he advises his patients with gestational diabetes to control their blood glucose, many don't listen. They might agree in the moment, but they won't follow through because they feel it doesn't matter, and they're not experiencing any discomfort…” P18‐Psotpartum (2 m)‐attendee “I haven't felt any discomfort myself, so I think there shouldn't be any major issues”. P11‐Postpartum (1 yr)‐non‐attendee

#### 3.1.1 Risk awareness

Risk awareness of GDM and T2DM influenced women's participation in postpartum screening. A lack of awareness regarding the long‐term risks from GDM was common among women who did not attend screening. Some women assumed glucose levels would normalise after childbirth and did not realise the association between GDM and the risk of T2DM. This misconception reduced the relevance of postpartum diabetes screening for the women.

While some women understood the link between GDM and the risk of developing T2DM, they still did not feel motivated because they were unaware of the complications of T2DM. Conversely, women with a family history of diabetes who had observed its associated complications were more vigilant about their diabetes risk and attended the screening. The women who attended the screening suggested that in order to boost screening attendance, it is important to provide education to increase the risk awareness of T2DM after GDM and emphasise the benefits of postpartum diabetes screening.

#### 3.1.2 Interactions with HCPs

HCPs' prioritisation of the importance of screening and their attitude toward GDM influenced women's attendance at postpartum screening. Women who did not attend screening felt that their HCP did not emphasise the importance of it. Some women requested more information, which was ignored, and the risks and benefits of screening were unexplained. Some women perceived that this lack of interest in their postpartum health from HCPs was reflective of a general shift in focus from them to their infants.

The failure to provide the relevant follow‐up support specific to the screening outcomes was another potential barrier, as some women felt that a lack of such support after screening made it seem less meaningful. Conversely, women who felt their HCP was supportive were more willing to attend screening. These data suggest that HCPs' prioritisation of GDM risk and the importance of screening, characterised by scheduling the test before discharge, offering advice and providing comprehensive risk education to women, could greatly impact the uptake of screening.

#### 3.1.3 Screening method‐related factors

##### Understanding and beliefs of screening methods, procedures and timing

A lack of clarity on the different screening methods, procedures and timings of when it should occur influenced the uptake of postpartum diabetes screening. Some women expressed confusion about the purpose of consuming a sugary drink during the OGTT to assess glucose levels and questioned the validity of the test. They believed that consuming a sugary drink would just result in a high glucose level. Additionally, the term ‘glucose tolerance’ was not well understood by women, adding to their confusion.

There was a lack of understanding regarding the difference between the post‐childbirth glucose test and postpartum diabetes screening. Women who received a normal post‐birth glucose test during their hospital stay or those who self‐monitored their glucose levels at home were less inclined to attend postpartum diabetes screening, believing it was unnecessary.

The perceived negative impact of consuming a sugary drink was frequently mentioned by women who did not attend screening. They worried that consuming a sugary drink would harm them and their babies (via breast milk). Concerns about the amount of blood drawn as part of the OGTT also contributed to their reluctance.

Conversely, gaining knowledge about the screening method and acknowledging the accuracy of the OGTT test facilitated women's attendance. Some reported that understanding its benefits helped them overcome their aversion to consuming the sugary drink.

#### 3.1.4 Accessibility of the screening

According to women's accounts, attending postpartum diabetes screening is negatively influenced by childcare responsibilities, geographical distance, transportation challenges and financial concerns.

Women reported that competing priorities were a significant barrier to attending screening. This was mainly related to childcare responsibilities. An additional problem was having both the baby and mother's postpartum check‐ups on the same day as their diabetes screening when they had their babies with them. Women reported that support from families, particularly with childcare, was helpful.

Difficulties booking screening appointments were reported by some women. For example, they had to book the OGTT specifically in the endocrinology department rather than the obstetric clinic, where women typically receive most postpartum maternal check‐ups. The inconvenient testing location, coupled with transportation challenges and geographical distance, made accessing screening more difficult. This was particularly important among those living in rural areas who had to travel to urban centres to attend screening.

Costs were identified as a potential barrier, discouraging some women, especially those without medical insurance, from attending postpartum screening. Women suggested welfare support, particularly financial assistance for the cost of the screening, as a facilitator.

#### 3.1.5 Acceptability of the test

Many women reported poor acceptability of the screening method and shared negative experiences with an OGTT during their pregnancies. Common reasons for not wanting to attend included long waiting times, the unpleasant taste of the sugary drink and the requirement to fast. Some women suggested that offering alternative carbohydrates to the sugary drink might increase the acceptability of screening with an OGTT.

#### 3.1.6 Motivation to maintain personal health

Women's motivation to attend to their personal health needs, including postpartum diabetes screening, was influenced by several factors, including fear of T2DM and GDM diagnoses and their treatment requirements, the asymptomatic nature of hyperglycaemia and their perceived confidence in their current self management.

##### Fear

Fear functioned both as a barrier and a facilitator to postpartum GDM screening. While some women reported that fear of diabetes management, including diet control and finger pricking for capillary blood glucose monitoring, prevented them from attending the screening, others described fear as a motivator, driving them to attend the screening to reduce their risks.

##### Looking after their child

While some women paid attention to their condition during pregnancy for the sake of their baby, they perceived that GDM was not worrisome after childbirth, as their baby's health was no longer dependent on them regulating their blood glucose levels. In the postpartum period, some reported their major focus was on taking care of their newborn, leading to their own health being gradually neglected. This prioritization of the care and health of their newborn served as a barrier to attending postpartum diabetes screening.

However, other women indicated that the responsibility of motherhood prompted them to pay more attention to their own health, enhancing their motivation to attend.

##### Asymptomatic nature of hyperglycaemia

The absence of any symptoms from hyperglycaemia led some women to hold an optimistic perception of the risk of developing T2DM. This perception reduced their motivation to attend postpartum screening.

##### Perceived confidence in self care

Some women cited confidence in their ability to manage their own health as a reason for not participating in postpartum diabetes screening. They believed that managing their diet meant they did not need the screening as they were tackling the risk themselves and the test would not impact what they were doing.

## 4 DISCUSSION

This study explored barriers and facilitators of attending postpartum diabetes screening from the perspective of women with GDM in China. Four relevant themes were identified from the data, including risk awareness, screening‐related factors, interactions with HCPs, and motivation related to personal health. These findings provide important insights into what regulates women's behaviours toward screening. The theoretical framework (incorporating the TDF and COM‐B) identified from the data could be used to develop strategies and interventions to improve the uptake of postpartum diabetes screening in this high‐risk population for T2DM.

A key factor in women's screening behaviours was the lack of awareness of the long‐term impacts of GDM and the risk of T2DM, an observation that has been reported in previous studies.^30^, ^32^ The study findings showed a deficit in risk education on the value and importance of screening. Additionally, the intensity of education provided during the GDM pregnancy, at a time when women face multiple demands and may feel overwhelmed, was found to be less ideal. The study also identified a decreased focus on personal health among women in the postpartum period, as they believed their newborn's health was no longer dependent on them. This suggests that risk information and support need to be provided both antenatally, when women are more receptive, and postpartum, when women tend to prioritise newborn care over their own health. Women also need to gain a better understanding of the health risks associated with T2DM.

The lack of awareness regarding the long‐term impacts of GDM and the risk of T2DM observed in this study is congruent with findings from previous survey studies.^35^, ^44^ A cross‐sectional study of women with GDM comparing women who did or did not attend screening conducted in Saudi Arabia reported that being informed about the risk of high glucose levels was associated with screening uptake (OR:2.381, 95%CI:1.178–4.811).^45^ In a previous Bayesian network meta‐analysis of interventions to improve postpartum screening uptake, we found that antenatal education on screening was associated with improved uptake.^46^ Therefore, providing timely education on the need for screening in the context of diabetes risk awareness in a positive way both antenatally and in the postpartum period could be useful in improving the uptake of postpartum diabetes screening among Chinese women.

The study findings also showed how women's awareness of the risk of developing T2DM was influenced by their interactions with HCPs. If HCPs fail to explain the importance of screening in the context of the risk of diabetes, this can lead women to underestimate the importance of the screening. In addition, the women who did not attend screening reported that this was in part related to their HCPs dismissing their concerns and needs, which they felt was related to the HCP's shift in attention toward their care of the infant rather than them. Conversely, attentive care from HCPs, characterised by explaining the risks of developing T2DM and the importance of screening and scheduling the test before discharge, was important in facilitating women's participation in screening.

The importance of HCP interactions in the uptake of screening has been reported in previous studies,^29^, ^38^, ^39^ which suggested that receiving advice from HCPs regarding the purpose of screening and what it entailed increased screening uptake. A qualitative synthesis^47^ of studies related to postpartum screening from the HCPs perspective identified that a reluctance to communicate risks, lack of time and short‐term focused consultations, and lack of role clarity impeded the provision of education on diabetes risk and the importance of screening. Another mixed method systematic review study^48^ found that some HCPs considered postpartum diabetes screening a low priority, or they reported being too busy to provide adequate provision of postpartum care. This highlights the need to better equip and motivate HCPs to provide more consistent, constructive, and supportive risk education to women on screening in the context of the risk for future GDM or T2DM. The data from this study suggest this is equally important in China.

A related issue revealed in the study was the lack of consistent follow‐up care reported by the women who did not attend screening. This lack of follow‐up care experienced indicates a deficit in care consistency. One potential solution to this problem would be to establish a clear care pathway to ensure better continuity of care for women into the postpartum period that included access to diabetes screening. It would also be important to integrate individualised support and education on diabetes prevention into that pathway. It has also been suggested that integrating postpartum care across the maternity and primary care settings might improve the continuity and support provided to women in helping them reduce the risk of future diabetes.^49^, ^50^

Factors related to screening were among the most prominent influences on the uptake of screening. These factors included the accessibility and acceptability of the screening process and method, as well as women's understanding and beliefs about its purpose, and value to them. Many women in the study reported negative attitudes toward the requirements of the primary screening method, the OGTT. The need to fast, the long waiting time, venous blood sampling, and the unpleasant taste of the glucose solution were frequently mentioned as the factors contributing to the reluctance to undertake the screening test. These findings align with previous studies, which have found that the OGTT was associated with high levels of anxiety and physical pain, and a dislike for the glucose solution.^51^ Some women interviewed in this study suggested that providing an alternative glucose solution could increase the acceptability and reduce the discomfort during the test. It has also been shown that providing alternative screening methods (fasting glucose, HbA1c or at home capillary glucose self monitoring) can increase screening uptake.^46^ These data suggest that providing alternative glucose solutions or different glucose tests may improve women's engagement with postpartum screening. However, making changes to the screening process may be challenging in China as currently the OGTT is the only recommended screening method. If adaptations to screening processes or methods are undertaken, it is important to ensure that this does not diminish the sensitivity and specificity of the test.

The study findings also indicate that women's beliefs about the purpose and consequence of the test also influenced attendance at screening. This included confusion over how drinking glucose could be valid when they know glucose increases the amount of sugar in their blood. They were also confused by the idea of consuming a sugary drink when they had been told to avoid such drinks during their pregnancy. Some women also did not want to know the results of the test due to fear of a diagnosis with diabetes. To address these beliefs, women need a clearer explanation of the rationale for consuming a glucose solution, the meaning of glucose intolerance, the difference between post‐birth glucose tests and postpartum diabetes screening, and a constructive conversation on what the results of the test may indicate. These issues could be addressed through education that is more meaningfully oriented to the needs of the women and delivered in a way they can relate to.

Accessibility of the screening was identified as another factor influencing screening uptake, with travel, appointment scheduling and competing priorities with childcare all presenting barriers. Accessibility has also been highlighted in other studies as a barrier.^30^, ^33^, ^52^ Therefore, screening appointments need to be much more flexible in their timing, with options for more localized screening and held in child‐friendly spaces. Women may also need childcare support to attend. The cost of screening provided another barrier, especially among women who were uninsured. Hence, some assistance with costs was advocated by some participants.

The study found that women's motivation to attend screening was influenced by multiple factors. As reported in previous studies, the absence of any symptoms, a belief they could self‐manage their glucose levels, and fear of a T2DM diagnosis all reduced motivation to attend screening.^30^, ^32^, ^52^ Consistent with findings from previous studies,^33^, ^38^, ^53^ women in this study also reported their focus on personal health diminished as they prioritised attending to their newborn infant. These findings emphasise the need for a balanced approach to ensure women understand the risk of diabetes, receive positive information on preventing diabetes, and recognise their own health needs in the context of their infant and family health.

## 5 STRENGTHS AND LIMITATIONS

The following limitations need to be considered in respect of our study. The transferability of the findings may be somewhat limited as they were derived from a one country context, although many of the observations from these data did align with previous studies conducted elsewhere. Hence, despite this limitation, the findings may provide a useful framework to be considered in conjunction with the wider research literature for developing interventions to improve screening uptake. Another potential limitation was that the studies were conducted online, which could have influenced what women were prepared to say. However, the online interviews meant that women from different parts of China and those with busy childcare schedules could participate and voice their experiences, making these findings more transferable across those regions. Finally, there was a potential bias in the participants in that a high proportion had university education, although the profile of participants closely mirrors the age specific national education level in China.

Study strengths included having participants from both the antenatal and postpartum periods, giving insights from women who had been offered screening and those who would have to consider it after their delivery. This approach allows for a broader perspective on the barriers and facilitators to postpartum diabetes screening from across the perinatal continuum, recognising that perspectives are interconnected and shaped throughout this period. This study also involved a geographically diverse population across China. A further strength was the adoption of the TDF framework and COM‐B model to help formulate the study findings into a behavioural model, providing a framework to support future intervention development.

## 6 CONCLUSION

The findings from this study suggested that risk awareness, HCP interactions, screening‐related factors, and motivation to maintain personal health are key factors in screening uptake among women in China. These findings suggest important considerations to increase screening uptake in this population. Women need more risk awareness education, and effective HCP communication would be beneficial. Additionally, improving screening acceptability and accessibility and targeting the psychological determinants might also help to improve screening uptake.

## CONFLICT OF INTEREST STATEMENT

None.

## Supporting information


Appendix S1.
