# Supplementary material for: Barriers and facilitators to attending postpartum diabetes screening among women with previous gestational diabetes in China: A qualitative analysis
Source: Diabet Med. 2025 Apr 11;42(8):e70043. doi: 10.1111/dme.70043 (PMC12257430; doi:10.1111/dme.70043)
Supplement: Supplementary file 1 — Appendix S1. [file DME-42-e70043-s001.docx]

Appendix 1. Topic guide

- **Introduction**

1. Introduce the researcher and the purpose of the interview
2. Explain the interview procedure
3. Informed consent
4. Begin recording

[For both the screened group and unscreened group]

- **Understand current GDM follow-up care**

Please tell me a bit about your experience with current GDM follow-up care. What is the current follow-up care? What do you think of it?

Did you receive follow-up advice/suggestions from healthcare professionals after you gave birth?

How could current follow-up care be improved?

- **Identify needs during the postpartum period**

Please think about the 3 months/x year after you gave birth. What were the most important things to you during this time?

What kind of support do you want to receive during the postpartum period?

What are you most worried about after getting gestational diabetes? Especially during the postpartum period?

- **Views on postpartum diabetes screening**

Do you think that some women are at greater risk of developing T2DM?

What do you think of postpartum diabetes screening? Is it necessary to receive postpartum diabetes screening?

What is it like to attend postpartum diabetes screening? [For the screened group]

- **Barriers and facilitators to attending postpartum diabetes screening**

[For the screened group]

How long after you gave birth were you tested for diabetes? What test did you have?

What types of support made it easier/possible for you to get tested for diabetes after pregnancy?

How do you feel like having regular diabetes tests in the future?

[For the unscreened group]

What were some of the things that made it hard for you to get tested for diabetes? In what ways did they affect your ability to get tested for diabetes? Which of the things has the biggest impact on you?

- **Strategies to promote postpartum diabetes screening**

Please think about the time after birth, what your family, doctors, nurses, or others could have done to help you get tested for diabetes after pregnancy? /if you were the health centre director, what kind of things would you do to help women get tested for diabetes after pregnancy?
